# Nevoid basal cell carcinoma syndrome (Gorlin syndrome)

**DOI:** 10.1186/1750-1172-3-32

**Published:** 2008-11-25

**Authors:** Lorenzo Lo Muzio

**Affiliations:** 1 Dipartimento di Scienze Chirurgiche, Facoltà di Medicina e Chirurgia, Università degli Studi di Foggia, Foggia, Italy

## Abstract

Nevoid basal cell carcinoma syndrome (NBCCS), also known as Gorlin syndrome, is a hereditary condition characterized by a wide range of developmental abnormalities and a predisposition to neoplasms.

The estimated prevalence varies from 1/57,000 to 1/256,000, with a male-to-female ratio of 1:1.

Main clinical manifestations include multiple basal cell carcinomas (BCCs), odontogenic keratocysts of the jaws, hyperkeratosis of palms and soles, skeletal abnormalities, intracranial ectopic calcifications, and facial dysmorphism (macrocephaly, cleft lip/palate and severe eye anomalies). Intellectual deficit is present in up to 5% of cases. BCCs (varying clinically from flesh-colored papules to ulcerating plaques and in diameter from 1 to 10 mm) are most commonly located on the face, back and chest. The number of BBCs varies from a few to several thousand. Recurrent jaw cysts occur in 90% of patients. Skeletal abnormalities (affecting the shape of the ribs, vertebral column bones, and the skull) are frequent. Ocular, genitourinary and cardiovascular disorders may occur. About 5–10% of NBCCS patients develop the brain malignancy medulloblastoma, which may be a potential cause of early death.

NBCCS is caused by mutations in the *PTCH1 *gene and is transmitted as an autosomal dominant trait with complete penetrance and variable expressivity.

Clinical diagnosis relies on specific criteria. Gene mutation analysis confirms the diagnosis. Genetic counseling is mandatory. Antenatal diagnosis is feasible by means of ultrasound scans and analysis of DNA extracted from fetal cells (obtained by amniocentesis or chorionic villus sampling). Main differential diagnoses include Bazex syndrome, trichoepithelioma papulosum multiplex and Torre's syndrome (Muir-Torre's syndrome).

Management requires a multidisciplinary approach. Keratocysts are treated by surgical removal. Surgery for BBCs is indicated when the number of lesions is limited; other treatments include laser ablation, photodynamic therapy and topical chemotherapy. Radiotherapy should be avoided. Vitamin A analogs may play a preventive role against development of new BCCs.

Life expectancy in NBCCS is not significantly altered but morbidity from complications can be substantial. Regular follow-up by a multi-specialist team (dermatologist, neurologist and odontologist) should be offered. Patients with NBCCS should strictly avoid an excessive sun exposure.

## Disease name and synonyms

Nevoid basal cell carcinoma syndrome (NBCCS)

Gorlin syndrome

Gorlin-Goltz syndrome

Basal cell nevus syndrome, BCNS (OMIM 109400)

Fifth phacomatosis

## Definition

The syndrome, delineated by Gorlin and Goltz in 1960 [1], was first reported in 1894 [2,3] and has been given several different names:

• Gorlin syndrome

• Gorlin-Goltz syndrome

• Nevoid basal cell carcinoma syndrome (NBCCS)

• Multiple nevoid basal-cell carcinoma syndrome (MNBCCS)

• Multiple basal-cell carcinoma syndrome

• Basal cell nevus syndrome (BCNS)

• Multiple basalioma syndrome

• Fifth phacomatosis

• Jaw cysts-basal cell tumors-skeletal anomalies syndrome

• Odontogenic keratocytosis-skeletal anomalies syndrome

• Multiple nevoid basal-cell epithelioma-jaw cysts-bifid rib syndrome

• Hereditary cutaneomandibular polyoncosis

• Epitheliomatose multiple generalisee

Currently, this disorder, resulting from mutations in the *PTCH1 *gene, is called the ***nevoid basal cell carcinoma syndrome (NBCCS)***, as Professor Gorlin suggested. However, patients and their relatives prefer to use the name "***Gorlin syndrome***", since it does not contain the word "carcinoma", even if about 50% of white patients will develop a significant number of skin cancers. Skin cancers do not generally occur in black patients [4].

Diagnostic criteria, based on the most frequent and/or specific features of the syndrome, were defined by Evans *et al*. in the Journal of Medical Genetics in 1993 [5], and modified by Kimonis *et al*. in 1997 [6].

## Epidemiology

This syndrome existed during Dynastic Egyptian times as shown by a constellation of findings compatible with the syndrome in mummies dating back to 1,000 b.c. [7]. In 1992, Farndon *et al*. estimated that the minimum prevalence is 1 per 57,000 [8,9]. An almost identical value was noted by Pratt and Jackson [10]. A study in the North West of England showed that the disease affects 1 in 55,600 people [5]. In Italy, the incidence (1/256,000) [11] of the disease appears to be lower than in Australia (1/164,000) [12] and in the United Kingdom [5] (Table 1). Rahbari and Mehregan noted that 2% of patients under 45 years of age with basal cell carcinomas have the syndrome [13]. Recently two new studies reported the incidence of the syndrome in Korea [14], and in France[15].

**Table 1:** Comparison of prevalence and frequency of major criteria in NBCCS patients among four studies.

	Evans *et al*. [5] United Kingdom, 1993	Shanley *et al*. [12] Australia, 1994	Kimonis *et al*. [6] United States, 1997	Lo Muzio *et al*. [11] Italy, 1999	Ahn SG *et al*. [14] Korea, 2004	Pruvost-Balland *et al*. [15] France, 2006
Patients	84	118	105	37	33	22

Prevalence	1/56,000	1/164,000	NR	1/256,000	1/13,939,393	NR

Major criteria % or (% African-Americans)						

BCCs	47	76	80 (38)	30	15.2	100
Age > 20 y	73	85	91	17	-	-
Age > 40 y	90	95	97	75	-	-

Jaw cyst	66	75	74	92	90.9	62

Palm/plant pits	71	80	87	35	66.7	45

Calcification of falx cerebri	NR	92	65	70	21.2	66

NBCCS = Nevoid basal cell carcinoma syndrome; NR-Non reported

## Clinical description

Many different signs and symptoms may be associated with NBCCS (Table 2). Clinical manifestations include basal cell carcinomas (BCCs), which may appear very early (2 years of age) [16]; odontogenic keratocysts (OKCs) that generally develop in the first, second and third decades [6,11,12,17,18]; palmar and/or plantar pits, and ectopic calcifications of the falx cerebri. These manifestations are considered as the major criteria for diagnosis [5]. The disorder is also characterized by congenital skeletal abnormalities, macrocephaly with frontal bossing, cleft lip and/or palate, and eye anomalies. Various low-frequency neoplasms, such as medulloblastomas, meningiomas, and ovarian and cardiac fibromas have been also reported (Table 2).

**Table 2:** Disorders reported in patients with basal cell nevus syndrome.

**Clinical manifestations**	
**Skin**	**Central nervous system**
Cutaneous dyskeratosis:	Ectopic calcification:
∘ erythematous-squamous spots degenerating in NBCC	∘ falx cerebri
	∘ tentorium cerebelli
∘ nodular or patch lesions	∘ spotted meningeal calcification
∘ palmo-plantar pits	∘ complete or partial bony bridging of the sella turcica
Multiple basal cell carcinomas	
Benign dermal cysts	Meningioma
∘ Multiple nevi	Medulloblastoma
	Multiform glioblastoma
	Moderate mental retardation
	Grand mal
	Congenital hydrocephalus
	Huntington's chorea

**Stomatologic system**	**Ocular system**
Odontogenic keratocysts	Cataract, coloboma, microphthalmia
Dental ectopy, heterotopy	Chalazions
Impacted teeth	Internal strabismus
Dental agenesis	Rotatory nistagmus
Malocclusion	Exophthalmus
Maxillary fibrosarcoma	Hypertelorism
Ameloblastoma	Congenital blindness
Odontogenic myxoma	
Spindle cell carcinoma	
Cleft palate and lip	
Mandibular prognathism	
High-arched palate	
Squamous cell carcinoma	
Skeletal open bite	
Idiopathic pseudocyst	
Hyperplasia of the mandibular coronoid processes	

**Musculo-skeletal system**	**Cardio-vascular system**
Congenital skeletal anomalies:	Cardiac fibroma (interventricular septum)
∘ bifid, fused, splayed, or missing ribs	Absent internal carotid artery
∘ bifid wedges fused vertebra	
∘ scoliosis	
∘ frontal, temporal and parietal bossing	
∘ polydactyly	
∘ sindactyly	
∘ short fourth metarcapal	
∘ sprengel shoulder	
Polyostotic bone cysts	

**Genito-urinary system**	**Auditory system**
Males:	Middle ear anomalies
∘ hypogonadism	∘ otosclerosis
∘ cryptorchidism	∘ conductive hearing loss
Females:	Posteriorly angulated ears
∘ ovarian calcifications	
∘ ovarian cysts	
∘ ovarian fibroma	
hypogonadism	

**Respiratory system**	**Biochemical findings**
Bronchogenic cysts	High levels of CAMP
Hyaline membrane disease	High levels of AP

**Gastro-enteric system**	
Linfomesenteryc cysts	
Gastric polyps	

### Skin

*Basal cell carcinomas *may arise in various stages of the syndrome: most often they appear between puberty and 35 years of age [16], but cases have been reported in 3 or 4 year-old patients [5,12]; in general, the mean age of onset is about 25 years of age. The incidence varies widely among ethnic groups: only about 40% of black patients affected by NBCCS manifest BCCs, while in whites they are reported in up to 90% of cases. However, the incidence in whites is also variable with some studies showing a lower prevalence (30%), probably due to protective skin pigmentation [11], as already established for African-American patients [6,19]. BCCs may vary in number (from a few to literally thousands) and range in size from 1 to 10 mm in diameter. Clinically, they may vary from flesh-colored papules to ulcerating plaques and may be mistaken for skin tags, nevi, or hemangiomas. There appears to be a relationship with increased sun exposure, and therefore BCCs are most likely to appear on the sun-exposed parts of the body, but non-exposed areas may also be involved. The most common locations are the face, the back, and the chest; the waist and extremities are rarely involved; BCCs have also been described on genitalia [20]. BCCs in this syndrome behave in the same manner as sporadic BCCs. In general, it is after puberty that the basal cell cancers become aggressive and invade locally [5]. If a lesion increases in size or begins to bleed or crust, invasion should be suspected and excluded. Radiation therapy must be avoided [6,21], since tumors may appear in the radiation fields even after many years [16]. Evidence of metastatization has been rarely reported [22,23]. If left untreated, BCCs can lead to disfigurment, especially on the face. Wang and Goldberg reported a patient with NBCCS who developed multiple *polypoid basal cell carcinomas *(PBCC) in the perineum, leading to the recommendation that the perineum, perianal, and genital areas should be included in the routine examination of patients with NBCCS [24]. The histopathology of nevoid BCC cannot be differentiated from that of ordinary sporadic BCC [4]. The tumors are composed of nests and islands or sheets of large, deeply-stained nuclei with indistinct cell membranes [4]. At the periphery of each lesion, the epithelial cells are well-polarized, suggestive of cutaneous basal cells. About 30% of NBCCS patients have two or more types of BCC patterns (morphea-like, solid, superficial, cystic, adenoid, fibroepithelial) [4]. Commonly (30–50% of cases), there are milia intermixed with the basal cell islands and calcified foci may be noted, not only within the tumor but also in normal skin [4]. Perhaps this reflects the "turning on" of the bone morphogenetic protein (*BMP*) gene [4].

Between 30% and 65% of patients with the syndrome have small asymmetric *palmar-plantar pits *by the age of 10 years, this percentage rising to 80% by the age of 15 [21,25]. Pits occur in 85% of patients over the age of 20 years [25]. These lesions are 2–3 mm in diameter and 1–3 mm in depth [26], with the color of the base being red in caucasians and black in africans. They increase in number with age, are permanent, and are a strong diagnostic indicator whenever found in a child. They are found more commonly on the palms (77%) than on the soles (50%). The pits may be easier to see if the hands are soaked in warm water for about 10 minutes. No BCC has been found to arise from these pits [21].

*Multiple nevi *are present in 30–50% of patients under 20 years of age, rising to 70% in patients over the age of 20 years. The nevi are flesh colored, reddish brown or pearly, and the groups resemble moles, skin tags, an ordinary nevus, cell nevi or hemangiomas. Some of them grow rapidly for a few days (to a few weeks), then mostly remain static.

Other skin signs are *nodular or patch lesions*, and *benign dermal cysts*. Small keratin-filled cysts (*milia*) can be found on the face in 30% of cases, most commonly in the area below the eyes, but they can also occur on the forehead.

*Epidermoid cysts *occur on the limbs and the trunk in over 50% of cases. They are usually 1–2 cm in diameter and are particularly common around the knee. Skin tags are especially common around the neck; histopathology reveals the typical features of a basal cell carcinoma, but the skin tags do not generally change in size or shape.

Recently, Wilson *et al*. reported the occurrence of *hairy skin patches *(discrete patches of unusually long pigmented hair) on the skin of three patients affected by Gorlin syndrome from two unrelated families with confirmed heterozygous mutations in the *PTCH1 *gene, suggesting that the patches may represent a sign associated with the syndrome [27].

### Stomatologic system

Recurrent jaw cysts are the main oral sign, being present in 90% of patients [11]. These cysts are known as *keratocysts *as they are characterized by a thin external fibrous capsule and an internal lining of keratinized stratified squamous epithelium. Keratocysts appear in the tooth-bearing areas of the jaws, and are believed to arise from the dental lamina [21]. Cysts may appear in both jaws. In several cases, the first symptom is either an odontogenic keratocyst or an odontogenic cyst of the jaws [11,18]. Odontogenic keratocysts (OKCs) are the most consistent and representative signs of NBCCS in the first and the second decades of life. OKCs are detected in patients under 10 years of age [5,12]: 13% of patients develop a jaw cyst by the age of 10 years and 51% by the age of 20 years. These data suggest that lesions may start to develop as early as at 7 or 8 years of age and underline the fact that keratocysts in patients affected by NBCCS arise earlier than those in healthy subjects [28,29]. In NBCC patients, there is continued development of new and recurring cysts until about age 30, when the rate of development tends to decrease [30]. Most non-NBCCS keratocysts are isolated lesions, but in NBCCS the keratocysts are commonly multiple, from 1 to 30, and the average number is 5. The most common site in the mandible is the molar-ramus region (44%), followed by the incisor-canine region (18%) [18]. In contrast, the majority of maxillary cysts occur in the incisor-canine (15%) and molar-tuberosity (13%) regions [18]. Only a few cysts have been reported in the premolar region. There is no racial predilection. There may be remarkably few symptoms until cysts reach a large size, especially when the ascending ramus is involved. Presentations include swelling and/or displaced, impacted teeth. About one-half of patients present with swelling, a quarter with mild pain, and 15% with an unusual taste following rupture of a cyst. Despite these manifestations, jaw fractures almost never occur. Radiographically, the keratocyst may show a unilocular or multilocular pattern and the cystic spaces may have a smooth or scalloped border. One of the most problematic aspects is the 60% rate of recurrence following surgery. In part, this may be due to incomplete removal, *i.e.*, from retention of epithelial islands and/or satellite microcysts, which occur with great frequency in the connective tissue capsule, or from proliferation of the basal layer of the epithelium [31]. There have been a few reports of ameloblastoma arising in odontogenic keratocysts [32] or, rarely, of squamous cell carcinoma [33]. Microscopically, the odontogenic keratocysts are multilocular with a parakeratinized (96%) or, rarely, orthokeratinized (4%) stratified squamous epithelium consisting of only a few layers of cells (about five to eight) with a well-defined basal epithelial cell layer, palisaded nuclei but no rete ridges [4]. Budding of the epithelium into the connective tissue with suprabasilar splitting has been noted in at least 50% of cases [4].

Other defects of the stomatologic system are malocclusion, impacted teeth, mandibular prognathism, dental ectopy or heterotopy, and dental agenesis. Cleft palate and lip are rare (5%) [34,35]. Recently, a new sign has been described: bilateral hyperplasia of the mandibular coronoid processes [36], which may be a radiologic criterion for the syndrome, useful for establishing a diagnosis especially in pediatric patients [37].

### Ectopic calcification of the central nervous system and other brain signs

Ectopic calcifications of the central nervous system have been reported: *lamellar calcification of the falx cerebri *(70–85%) [11], *calcification of the tentorium cerebelli *(20–40%) [11], *the petroclinoid ligament *(20%) and *the diaphragma sellae *(60–80%) [38], and *complete or partial bony bridging of the sella turcica *(25%) [11]. Spotted meningeal calcifications are rare. These signs may not result in clinical manifestations, but can be useful in confirming the diagnosis. The calcification of the falx cerebri can appear very early in life, and is often strikingly apparent from late childhood. Other signs reported are cysts of the choroid plexus of the third and lateral ventricles of the brain, intraparenchymal brain cysts, arachnoid cysts, glial nodules projecting from the lateral ventricles, agenesis of the corpus callosum with or without lipoma [39], "empty" sella syndrome [39], communicating hydrocephalus [40-43], meningioma [44,45], cysts of the septum pellucidum [46] and medulloblastoma [44,47-55] (see the paragraph "Other tumors"). Vermian dysgenesis has been noted in a mother and a daughter [56,57]. Seizures have occasionally been noted unassociated with brain tumors and possibly due to focal neuronal heterotopia [58]. Mental retardation occurs in about 5% of cases.

### Skeletal system

Skeletal manifestations are also present in NBCCS [46]. The signs in the bones found on X-ray may be helpful in making the diagnosis. The height (these patients can be much taller than expected for their family), the shape of the ribs, the shape of the bones in the vertebral column, and the shape of the skull are principally affected [46,59]. Mean height is increased (183 cm in males, 174 cm in females) and about 15% of patients are extremely tall [4]. An abnormal skull configuration (characterized by frontal (25%), biparetial or temporal bossing, and large calvaria) is frequent (70%) [11]. Relative macrocephaly with an occipital frontal circumference above the 95^th ^centile has been reported in 50% of cases [6,59]. As a result of the abnormal growth of the skull (> 60 cm in adults), about 70% of patients affected by NBCCS have eyes which appear wider apart than usual. Other skeletal signs are scoliosis (40%), and abnormalities such as bifid, wide, fused, partially missing or underdeveloped ribs (30–60%) [11]. Rib abnormalities may give rise to a prominent or depressed sternum in about 30% – 40% of patients. Spina bifida occulta of the cervical or thoracic vertebrae is found in 60% of cases [60]. However, in a series studied by Kimonis *et al*., the spina bifida occulta occurred in only 20% of patients [6]. Bifid wedges, fused vertebra and Sprengel anomaly (elevation of the scapula with rotation toward the spine with scoliosis) are observed in 10 to 40% of patients. Extra digits on the hands or feet can occur. In about 35% of individuals with Gorlin syndrome, an X-ray of the hands will show small cysts in the bones of the fingers. The numbers and site can be very variable. Bone cysts can also occur in the long bones of the arms and legs, the pelvis and the calvaria [61]; usually, these do not cause problems, even if calvarial involvement may give the erroneous impression that medulloblastoma has spread to bone [62]. Histologically, the flame-like lesions consist of fibrous connective tissue, nerves, and blood vessels, *i.e.*, they are hamartomas [4].

### Ocular system

NBCCS patients may have various ocular problems that occur with a frequency higher than that in the normal population (20%). These include hypertelorism (70%), exophthalmus, rotatory nystagmus, internal strabismus, congenital cataracts, orbital cysts, coloboma of the iris, choroid, and optic nerve, microphthalmia, and chalazions [5,11,21,63]. Most striking are small very transient keratin-filled cysts (milia) found on the palpebral conjunctivae in about 40% of cases [4]. This observation is a personal one, reported by a parental support group in Manchester, England [4]. The author asked the assembled group if they had ever had one or more of these transient cysts [4], and this exercise was repeated for the American support group for NBCCS [4]. Due to the transient nature of these cysts, most of the patients noted that they were not significantly disturbed by their presence, but they had been to ophthalmologists who confirmed the presence of milia of the palpebral mucosa [4].

### Genito-urinary system

Ovarian cysts and fibromas are present in 25–50% of affected women [5] and are often bilateral (75%). Ovarian calcifications are reported and may be mistaken for calcified uterine fibroids or calcified uterine leiomyomas. They do not seem to reduce fertility but may undergo torsion (twist). Some cases of ovarian fibrosarcoma [64] and primary ovarian leiomyosarcoma [65] have been reported. Males may exhibit hypogonadotrophic hypogonadism (5–10%), cryptorchidism, female pubic escutcheon, or gynecomastia. Other signs involving the kidneys (5%) may be: horseshoe kidney, L-shaped kidney, unilateral renal agenesis, renal cysts, or duplication of the renal pelvis and ureter.

### Mesenteric cysts

Single or multiple chylous or lymphatic mesenteric cysts have been documented [4,66-68]. The cysts are thin-walled and measure 2 to 14 cm in diameter, do not produce symptoms, and are often an occasional finding [21]. The content is chylous but may contain hemorrhagic turbid fluid [4]. Microscopically, the wall is composed of fibrous connective tissue and smooth muscle, and a layer of lymphatic cells is often located beneath the endothelial lining [4].

### Cardiovascular system

Fibroma of the heart has been described [5,21,46,69-74]. The syndrome is present in 3–5% of all patients with a cardiac fibroma [5,69]. The cardiac fibroma is discrete, well-circumscribed, not encapsulated, firm, gray-white, 3 to 4 cm in diameter, and may, upon occasion, exhibit central calcification [4]. The tumor is most often located in the left anterior ventricular wall. If the tumor projects into the chambers, hemodynamics is altered and/or impeded. Conduction defects (*i.e.*, arrhythmias) may also be present due to involvement of the intraventricular septum [75]. The tumors are composed of fibroblasts embedded in a dense matrix of collagen and elastic fibers.

### Other tumors

Tumors in patients with NBCCS include basal cell carcinoma, medulloblastoma, fibroma and rhabdomyosarcoma (RMS) [76-78]. RMSs are also present in 15% of mice haplodeficient for *PTCH1 *[79]. *Medulloblastoma *is frequent [44,47-55]; Herzberg and Wiskemann first pointed out the association of NBCCS with medulloblastoma in 1963 [80]. The tumor characteristically presents during the first two years of life, while in the general population occurrence peaks at 7 to 8 years of age. The incidence of medulloblastoma in Evans' series was 1% to 2% [5]. There is a sex predilection with a male-to-female ratio of 3:1. Early onset medulloblastoma may be the presenting sign of NBCCS; thus, in children in which this tumor is diagnosed, NBCCS should be suspected and a careful examination for other signs of the syndrome should be performed in order to rule it out [6]. There is some evidence that medulloblastomas associated with NBCCS have a better clinical outcome than those found in isolation [47,81,82], with the possibility of spontaneous recovery [82]. Radiation therapy for medulloblastoma can induce invasive BCCs in the radiation field (*i.e.*, from the nape to base of the spine) [83].

Other brain tumors, such as *astrocystoma, craniopharyngioma*, and *oligodendroglioma*, are infrequent, while *meningioma *is slightly more common. These tumors may well be secondary to radiation therapy.

In 1968, Schweisguth *et al*. reported *fetal rhabdomyoma *[84]. This tumor has been described at various sites [21,78,85-89]. Mature striated muscle within the tumor is indicative for diagnosis.

There are several reports that support an increased incidence of other neoplasms or hamartomas, such as leiomyomas of bowel and mesentery, leiomyosarcoma, adrenal cystic lymphangioma, thyroid adenoma, lymphangiomyoma, melanoma, ameloblastoma, craniopharyngioma, mesenchymoma, Hodgkin's lymphoma, non-Hodgkin's lymphoma, seminoma, paratesticular pseudotumor, schwannoma, pleiomorphic adenoma of parotid, adenoid cystic carcinoma of salivary gland, and adrenal tumors [90-92].

## Etiology

NBCCS is a hereditary condition caused by mutations in the *PTCH1 *gene and is transmitted in an autosomal dominant manner with high penetrance and variable expressivity [93,94]. There is no sexual predilection. Individuals with no known affected family members may comprise up to 60% of all affected individuals [95].

The *PTCH1 *gene has recently been mapped to the long arm of chromosome 9 (q22.3-q31) with no apparent heterogeneity [8,96]. Approximately 50% of NBCCS patients have allelic losses including this site [97]. Data suggest that the product of this gene acts as a tumor suppressor [96,98,99]. Furthermore, the typical malformative pattern of NBCCS suggests that the gene has a fundamental function in controlling growth and development of normal tissues [100]. This gene was isolated in 1996 as the human homolog of the Drosophila *PTCH1 *gene, simultaneously in Australia and in the USA [101,102].

The *PTCH1 *gene in the fruit fly is important for body segmentation. If the gene is faulty, abnormal segmentation occurs. Several mutations of the *PTCH1 *gene have been identified in patients with NBCCS [101-118], and, in those with BCCs and medulloblastomas that are apparently not hereditary [119,120].

The gene consists of 23 exons, which span 34 kb. It encodes a transmembrane glycoprotein composed of 1447 amino acids, with 12 transmembrane domains and two large hydrophilic extracellular loops where Sonic Hedgehog (SHH) ligand binding occurs. If a mutation occurs in *PTCH1 *and the second loop is not formed, SHH cannot bind.

Thus, the *PTCH1 *gene product acts as membrane receptor [121] for the SHH signal and represses transcription (in certain cells) of genes encoding signaling proteins belonging to the transforming growth factor (TGF)-beta and Wnt families. The Hedgehog (Hh) family comprises several regulation factors described in several species. SHH is a true signaling switch used in differentiating subpopulations of cells throughout the embryo; in fact, SHH patterning is involved in several embryonal processes, such as the formation of the ventral neural tube (basal plate, motor columns), the limb bud, parts of the brain, hair follicle development, and tooth development. PTCH1 in the absence of its ligand, SHH, acts a cell-cycle regulator, normally inhibiting expression of downstream genes that control cell fate, patterning, and growth [122]. In the case of a C-terminal truncation caused by a mutation in *PTCH1*, these genes are no longer repressed.

The molecular biology of cancers in NBCCS is similar to that of retinoblastoma, the prototypical pediatric cancer syndrome, which is characterized by mutations in a recessive oncogene. Data from patients with retinoblastoma support the Knudson two-hit hypothesis; this theory of oncogenesis states that normal cells require two mutagenic hits (two distinct episodes of DNA damage) to produce a cancer. Patients with retinoblastoma, NBCCS, and other similar syndromes have a constitutional (germline) defect in the DNA sequence in one of the two copies of a tumor suppressor gene. A defect in one of the two copies is insufficient to cause a cancer. If a second DNA injury or if loss of the normal remaining allele occurs at the same locus (the second hit), the cell may become malignant. In NBCCS, various tumors and hamartomas (BCC, OKCs, meningiomas, ovarian fibromas) exhibit loss of heterozygosity [123,124], but other lesions do not, for example, palmar pits [125]. Loss of heterozygosity at the *PTCH1 *locus has been observed in almost 90% of hereditary BCCs. Various physical anomalies (bifid rib, macrocephaly) apparently need only one hit. An interesting aspect of this syndrome is that some of the developmental defects (*e.g. *jaw cysts) appear to have two genetic hits but without developing into a true malignancy. In some patients, the lining of these odontogenic keratocysts has lost the normal gene for NBCCS, while retaining the mutant allele. Agaram *et al*. found that 70% of jaw cysts showed loss of heterozygosity of one or more of the several common tumor suppressor genes [126]. This finding may explain the behavior of jaw cysts, which may be more similar to low-grade neoplasms than congenital malformations.

Analysis of gene mutations in the syndrome has identified deletions, insertions, splice site alterations, nonsense and missense mutations, with no hot spots identified for mutations. About 70% of germ-line *PTCH1 *mutations are rearrangements. Over 80% of these mutations seem to result in a truncation of the coded protein [115], which suggests that developmental anomalies seen in NBCC syndrome may be due to haplo-insufficiency [127]; recently, *PTCH1 *mutations have been reported as a result of errors in the recombinational repair process [109].

In 2006 Lindström *et al*. analyzed 284 mutations and 48 single nucleotide polymorphisms (SNPs) located in the *PTCH1 *gene, compiled from a *PTCH1 *mutation database [110]. They found that the *PTCH1 *mutations were mainly clustered into the two large extracellular loops of the corresponding protein and its large intracellular loop [110]. The SNPs appeared to be clustered around the sterol-sensing domain and the second half of the protein [110]. NBCCS cases and each class of tumor analyzed revealed a different distribution of the mutations in the various *PTCH1 *domains [110]. Moreover, the types of mutations were also unique for the different groups [110]. Finally, the *PTCH1 *gene harbors mutational hot spot residues and regions, including a slippage-sensitive sequence in the N-terminus [110].

Several studies reported polymorphisms for the *PTCH1 *gene in North American [128], European [115,129], Chinese [130], and Japanese [131] populations. Some of these polymorphic variants (*i.e*., 1686C>T, 2199A>G, 3944T>C, IVS10-51G>C, IVS10-8T>C) occur with a high frequency. A recent study has reported that certain haplotypes of *PTCH1 *polymorphisms may mediate the susceptibility to basal cell carcinomas [132]. Pedigrees with multiple features of NBCCS were most likely to test positive for *PTCH1 *mutations [133]. Pedigrees with multiple or early-onset basal cell carcinomas without other features of the disease did not test positive for *PTCH1 *mutations [133].

*PTCH2*, highly homologous to *PTCH1*, has been mapped to chromosome 1p32.1-p32.3 [134]. *PTCH2 *comprises 22 coding exons and spans approximately 15 kb [134]. It encodes a 1203 amino acid putative transmembrane protein that is highly homologous to the PTCH1 product. Mutations have been found in one simplex case (*i.e.*, a single occurrence of the disease in a family) of medulloblastoma and one simplex case of BCC [134]. No *PTCH2 *mutations were found in 11 simplex cases of NBCCS or in 11 individuals with familial cases of NBCCS who did not have identifiable *PTCH1 *mutations. In many cases, medulloblastoma arising from perturbations of PTCH1 function leads to a concomitant up-regulation of the highly similar gene *PTCH2*. Lee *et al*. investigated the role of PTCH2 in tumor suppression by generating *PTCH2*-deficient mice [135]. In striking contrast to *PTCH1-/- *mice, *PTCH2-/- *animals were born alive and showed no obvious defects and were not cancer prone [135]. However, loss of PTCH2 markedly affected tumor formation in combination with PTCH1 haploinsufficiency. *PTCH1+/-PTCH2-/- *and *PTCH1+/-PTCH2+/- *animals showed a higher incidence of tumors and a broader spectrum of tumor types compared with *PTCH1+/- *animals [135]. Therefore, *PTCH2 *modulates tumorigenesis associated with PTCH1 haploinsufficiency [135].

## Diagnostic methods

Diagnosis of NBCCS can be made in the presence of two major criteria or one major and two minor criteria [6]:

### Major criteria

1. Multiple (>2) BCCs or one under 20 years

2. Odontogenic keratocysts of the jaws proven by histopathology

3. Palmar or plantar pits (3 or more)

4. Bilamellar calcification of the falx cerebri

5. Bifid, fused or markedly splayed ribs

6. First degree relatives with NBCCS

### Minor criteria

1. Macrocephaly determined after adjustment for height

2. Congenital malformation: cleft lip or palate, frontal bossing, "coarse face", moderate of severe hypertelorism

3. Other skeletal abnormalities: sprengel deformity, marked pectus deformity, marked syndactyly of the digits

4. Radiological abnormalities: bridging of the sella turcica, vertebral anomalies such as hemivertebrae, fusion or elongation of the vertebral bodies, modeling defects of the hands and feet, or flame-shaped lucencies of the hands or feet

5. Ovarian fibroma

6. Medulloblastoma.

The presence of the most frequent and/or specific features of the syndrome make the diagnosis easier; however, diagnosis in a child with a 50% risk of having inherited the condition may not be so easy because of the extreme variation in expression, both within and between families. Some children may have only rib anomalies, whilst others have the "typical face" without other signs. Gene tracking and mutation analysis may be helpful for pre-symptomatic diagnosis.

For apparently isolated cases, detailed examination and X-ray investigation of the relatives should be undertaken before concluding that a child's condition is the result of a new mutation. If an adult has no sign, no pertinent history and normal radiology, it is unlikely that he or she has Gorlin syndrome. Direct mutation analysis of the gene may be helpful. It is particularly helpful to follow a specific clinical protocol in the examination of these subjects (Table 3).

**Table 3:** Diagnostic protocols in NBCCS.

**Family history**
∘ past medical and dental history
**Clinical examinations**
∘ oral
∘ skin
∘ central nervous system
∘ head circumference
∘ interpupillar distance
∘ eyes
∘ genito-urinary system
∘ cardio-vascular system
∘ respiratory system
∘ skeletal system
**Genetic testing**
**X-ray**
∘ chest
∘ A.P. and lateral skull
∘ Panoramic radiograph
∘ Cervical and thoracic spine – A.P. and lateral
∘ Hands (for pseudocysts)
∘ Pelvic (female)
**Ovarian ultrasound (female) for ovarian fibroma**
**Echocardiogram (children) for cardiac fibroma**

In case of a family history of the disease, at birth or immediately after, a detailed examination should look for the presence of a large head, frontal and temporal bossing, cleft palate [35], eye anomalies and bifid ribs [16,19], or vertebral anomalies. In order to detect a deficit related to a medulloblastoma, a neurological examination is recommended every six months [5]. At 3 years, the frequency of examinations could be reduced to once a year until 7 years of age, after which a medulloblastoma is very unlikely.

A panoramic radiograph of the jaws once a year from the age of 8 years onward is also suggested [18]. The appearance of two or more recidivant OKCs, or an early OKC in a young subject, is suggestive of NBCCS syndrome. An at least annual examination of the skin from puberty is recommended but as a lesion may suddenly become aggressive, the patient needs open access to the specialist taking responsibility for treatment of the skin.

In children at risk, diagnosis can often be achieved by radiographic means (calcification of falx, rib anomalies, calcification of ovarian fibromas). Depending on the situation, imaging studies may include magnetic resonance imaging (MRI) of the brain, echocardiography, abdominal ultrasonography, dental radiography, and skeletal survey. Kimonis *et al*. described the radiologic findings that are most helpful in diagnosing NBCCS [46]. As patients with NBCCS are especially sensitive to ionizing radiation, the use of irradiation should be minimized when possible. Nevertheless, definitive presymptomatic diagnosis can be achieved by molecular genetic linkage. Rarely, babies can have fibromas in the heart: at least two cases have presented before 3 months of age with fibromas [71,136].

No specific laboratory studies are indicated; however, several laboratories offer commercial testing for Gorlin syndrome and testing is certainly indicated in children with desmoplastic medulloblastoma, where radiation is contemplated. Patients with suspected Gorlin syndrome should undergo biopsy with samples obtained from several suspicious skin lesions. The histologic features of BCCs in Gorlin syndrome are usually identical to those observed in sporadic BCCs. Medulloblastomas in Gorlin syndrome usually have the desmoplastic phenotype, which has been linked, in some studies, with an improved prognosis [47]. If an infratentorial mass is detected during brain MRI, it should be sampled during biopsy as well. The Chang staging system is used to stage medulloblastoma in Gorlin syndrome.

## Differential diagnosis

Precise differential diagnosis needs exclusion of some rare dermatological syndromes, such as Bazex syndrome (characterized by basal-cell carcinoma associated with hypotrichosis, hypohidrosis, milia and folliculare atrophodermia), trichoepithelioma papulosum multiplex (named also epithelioma adenoides cysticum), or Torre's syndrome (Muir-Torre's syndrome).

## Genetic counseling

The syndrome is a hereditary condition, thus referral to a geneticist for counseling is mandatory. NBCC syndrome is caused when one copy of the *PTCH1 *gene pair contains a fault; this means that every child of a person with the syndrome has a 1 in 2 (50:50) chance of inheriting the faulty gene. The risk to family members is various, about 70–80% of individuals diagnosed with NBCCS have an affected parent and about 20–30% of probands have a *de novo *mutation. The risk to a sib of a proband depends on the genetic status of the parents: if a parent of the proband is affected, the risk to the sibs is 50%; when the parents are clinically unaffected, the risk to the sibs of a proband appears to be low; if the disease-causing mutation cannot be detected in the DNA of the parent, the risk to sibs is low, but greater than that of the general population because of the possibility of somatic mosaicism or germline mosaicism exists. The offspring of an individual with mild NBCCS caused by somatic mosaicism may have a risk of less than 50% of inheriting the disease-causing mutation. The risk to other family members depends upon the genetic status of the proband's parents; if a parent is affected, his or her family members are at risk.

DNA tests, by looking directly at the mutation in the gene, may confirm that the patient is affected by this syndrome. In clinical situations suggesting genetic predisposition to BCC, germline mutations of *PTCH1 *are not common [107]. Genetic variants might contribute to the variation in numbers of BCCs and jaw cysts observed in NBCCS families [137].

It is recommended to involve families in regular screening. It may be possible to prove whether someone in a family has inherited the condition or not by DNA tests, either by tracking the chromosome 9 containing the faulty gene, or by direct analysis of the mutation causing the disease in a particular family.

In some families, only one person appears to be affected. This event is the result of two conditions: 1) the mother or the father have the syndrome, but the effects are so mild that it has never been diagnosed; 2) the fault in the *PTCH1 *gene has occurred for the first time in that family. It is a "new mutation" [16]. If a family member has not inherited the gene, the surveillance program is not necessary.

The definitive diagnostic test is to demonstrate a mutation in the *PTCH1 *gene, although this is labor-intensive as there are 24 exons. Recently Fujii *et al*. used newly designed high-resolution oligonucleotide microarrays with a median distance between the probes of 776 bp (average probe interval 2,271 bp) to detect gene deletions in NBCCS patients. This probe interval was chosen because small submicroscopic genomic deletions and duplications constitute up to 15% of all mutations underlying human monogenic diseases. Two out of three deletions could not be detected by conventional chromosomal analysis. A submicroscopic deletion as small as 165 kb was detected affecting only *PTCH1*, whereas the other two deletions were much larger (5 and 11 Mb) [138].

## Antenatal diagnosis

Antenatal diagnosis may be useful to prevent complications. Ultrasound scans during pregnancy may be helpful in detecting serious developmental malformations, even if they are rare. Some fetuses with NBCCS have large heads and so may need assistance in delivery either by forceps or by Cesarian section. Very rarely, fibroma of the heart may be detected. Prenatal diagnosis for pregnancies at increased risk is possible by analysis of DNA extracted from fetal cells obtained by amniocentesis (usually performed at about 15–18 weeks of gestation) or by chorionic villus sampling (CVS) at about 10–12 weeks of gestation. The disease-causing allele of an affected family member must be identified or linkage established in the family before prenatal testing can be performed. Requests for prenatal testing for conditions such as NBCCS that do not affect intellect, have variable expression and for which some treatments are available, are not common.

## Management including treatment

Patients affected by Gorlin syndrome must be treated by several specialists. Keratocysts can be treated by surgical removal. This requires exposure of the lesion by making an osteotomy in the jawbone under local or general anesthesia, finding the wall of the cyst and removing this completely. Cysts that recur after such a treatment are removed with a bone box including a layer of bone at the margin of the cyst. This is intended to ablate any daughter cysts; only in extreme cases it is necessary to remove an entire section of the affected jawbone.

As regards BCC, only a small fraction of these tumors become invasive and the very rare cases of death result from spread to the brain or lung. Results from several epidemiologic studies have indicated that the risks of BCCs show a strong positive correlation with exposure to UV radiation. Thus, these patients need to avoid excess sun exposure. They must use 100% UV protective sunglasses as the skin surrounding the eyes (similar to that of the nose/ears) is vulnerable to BCCs. Sunshine during the mid-day hours should be avoided if possible. High factor sunscreens (SPF30+) should be applied before going outside and re-applied every 2–3 hours, or more frequently, if perspiring or swimming.

Radiotherapy should be avoided if possible [6,21]. Tumors on the scalp can be particularly aggressive and need early intervention [5].

Management of superficial BCCs without follicular involvement can be accomplished by total body application of topical 0.1% tretinoin cream [16]. Patients should be examined every 3 months, and lesions manifesting growth must be excised [16]. More debatable is the use of oral retinoids or combined oral etretinate and surgical treatment [16]. Some authors indicated that doses of 0.5–1.0 mg/kg/day cause regression of lesions of less than 1.0 cm and prevent new lesions [139]. Nevertheless, a study of high-dose oral isotretinoin (mean daily dose: 3 mg/kg/day) found that only 8% of BCCs underwent complete clinical and histological regression, while all patients developed moderate-to-severe acute toxicity [140]. In addition, oral etretinate should not be taken by women of child-bearing age [16], a negative pregnancy test should be obtained prior to therapy and adequate contraception should be used by women during and for at least two months after stopping isotretinoin therapy.

Recent studies indicated that imiquimod 5% cream appears to be an effective treatment for nodular basal cell carcinoma alone [141-145], or if combined with curettage prior to its application [146]. In fact, 17 nodular BCCs on 15 patients were treated by curettage without electrodesiccation followed by once-daily application of imiquimod 5% cream, 5 times per week for 6 weeks; the area was excised and examined histologically 6 weeks after cessation of imiquimod cream and all 17 lesions (100%) showed no histological evidence of residual tumor on the post-treatment excision [146].

Surgical excision is the typical approach for a patient with BCC if the number of lesions is limited. Other treatments that may be effective and more efficient than surgical excision in patients with multiple lesions include laser ablation, photodynamic therapy, and topical chemotherapy [147].

Electrodessication and curettage is a very common procedure used as an alternative to topical chemotherapy in the treatment of small BCCs located in low recurrence areas of the body (neck, trunk, extremities) [148]. The area is first numbed with a local anesthetic injection and then scraped out from surrounding normal skin with a curette. An electrosurgical needle is then used to desiccate (heat and dry up) the remaining cancerous tissue. This is repeated for a total of three or four times in succession in order to achieve maximal cure rates. This form of treatment is quick, efficient and cost effective, but it is of limited value for large lesions and cancers of the mid face due to higher recurrence rates. Pain during treatment is minimal and post-operatively the patient feels nuisance comparable to a small burn. The cosmetic result will appear as a lighter hypopigmented flat spot that is of similar size as the cancer was prior to treatment. The chance of a definitive cure with this procedure is 92–93%. Another possibility is cryosurgery; it is quick, efficient and cost effective. However, this method should be avoided when treating lesions in high recurrence areas. The post-operative cosmetic result, follow-up care, and healing time is very similar to electrodessication and curettage. The overall chance of a cure with this procedure is 92.5%. Cryosurgery, in a few cases, was complicated by nerve damage and numbness, but in general has no side effects, except for scarring. Recently, laser vaporization with the carbon dioxide laser has been used alone or combined with curettage in cases of multiple/superficial tumors [149-151].

Surgical excision by scalpel allows removal of the cancerous tissue plus surrounding normal skin, with minimal discomfort during treatment and in the post-operative period. The cosmetic result is good, but it depends on the size and location of the tumor. The overall chance of a cure with surgical excision may range from 94–98%. Long term side effects include scarring, and rarely, nerve damage. The scalpel excision gives the advantage of being able to check the margins of the specimen microscopically by a pathologist.

Another therapeutic possibility is photodynamic therapy (PDT), a cancer treatment involving use of a photosensitizing dye (given intravenously or topically) that preferentially accumulates within malignant cells [16]. BCCs are then treated with red light (usually produced by a laser) that leads to dye activation and, definitively, to death of these cells [16]. Scabs form over the basal cell carcinomas; the scabs usually fall off by the end of the first month following PDT [16]. The management of patients with NBCCS consists of regular visits (every 2–3 months) to a dermatologist, especially during adolescence [16]. Recently, nodular BCCs resistant to multiple forms of treatment in two patients with NBCCS were treated with systemic porfimer sodium-based photodynamic therapy with a good response to treatment on clinical evaluation and on measurement by a 20-MHz high-resolution ultrasound [152].

Interferon has also been proposed in experimental studies for the treatment of BCCs. This substance is injected directly into the cancer three times each week, for three weeks. Some reports have shown complete resolution of treated tumors, but other studies demonstrated that this treatment was less effective [153]. Side effects include fever, chills, decreased white blood cell count, and pain at the site of injection. This procedure needs to be investigated further.

Patients with Gorlin syndrome can present with medulloblastoma, a malignant tumor of the posterior fossa [52]. Intensive multimodality therapy is typically required to treat medulloblastoma. The best outcomes are obtained in patients who are treated with aggressive resection, chemotherapy, and radiation therapy. Radiotherapy for medulloblastoma causes multiple BCCs in these patients [154,155] or intracranial and sinonasal tumors [83,156-158]; thus, post-operative radiotherapy should be used judiciously. This is particularly important in patients with the desmoplastic NBCCS subtype [47]. As a general rule, if possible, patients should be treated without radiation therapy or using a treatment field that only includes the posterior fossa [159], with a skin-sparing nonconformal technique [159]. In fact, the risk of multiple radiation-induced BCC is decreased with this technique that allows the exposition of skin to ionizing radiation to be reduced. On the contrary, this approach may increase the risk of other well-established toxicities, such as ototoxicity and irradiation of the temporal lobes.

## Prognosis

The most common lesions accounting for NBCCS are usually not life-threatening. Medulloblastoma may be a potential cause of early death, even if NBCCS patients may have better outcome than patients with sporadic medulloblastoma (as the desmoplastic subtype of medulloblastoma is associated with an improved survival compared to the classic form of medulloblastoma). Therefore, this could be one argument to limit therapy to young children with Gorlin syndrome and desmoplastic medulloblastoma. Problems can derive from some techniques used for the therapy of specific lesions: *i.e. *radiation therapy for medulloblastoma that may cause multiple BCCs. For this reason, nonstandard treatments should be considered in children with Gorlin syndrome and medulloblastoma. Chemoprevention may also be used to avoid skin lesions. Vitamin A analogs, such as retinoids, including isotretinoin, may play an important role in preventing or slowing the development of new BCCs [140,160-164].

## Support groups

A list of groups that support patients, families, and clinicians caring for patients with NBCCS is supplied in Additional file 1.

## Abbreviations

NBCCS: Nevoid basal cell carcinoma syndrome; MNBCCS: Multiple nevoid basal-cell carcinoma syndrome; BCNS: Basal cell nevus syndrome; BCCs: Basal cell carcinomas; OKCs: Odontogenic keratocysts; PBCC: Polypoid basal cell carcinomas; RMS: Rhabdomyosarcoma; SHH: Sonic Hedgehog; SNPs: Single nucleotide polymorphisms; MRI: Magnetic resonance imaging; PDT: Photodynamic therapy.

## Competing interests

The author declares that they have no competing interests.

## Supplementary Material

Additional file 1 A list of groups that support patients, families, and clinicians caring for patients with NBCCS. Click here for file
