# Supplementary material for: Nevoid basal cell carcinoma syndrome (Gorlin syndrome)
Source: Orphanet J Rare Dis. 2008 Nov 25;3:32. doi: 10.1186/1750-1172-3-32 (PMC2607262; doi:10.1186/1750-1172-3-32)
Supplement: Additional file 1 — A list of groups that support patients, families, and clinicians caring for patients with NBCCS. [file 1750-1172-3-32-S1.doc]

**Additional file 1.**

To date, there are some support groups in USA and in Europe: The Gorlin Syndrome Group, National Basal Cell Nevus Support and BCCNS Patient Advocate.

The Gorlin Syndrome Group was formed in the UK in 1992 by Mr Jim Costello, Founder and first Chair (now deceased), with the help of the Clinical Genetics Departments at St. Mary’s Hospital, Manchester and Birmingham Women’s Hospital, Professor GR Evans and Professor Farndon and their respective staff. (The Gorlin Syndrome Group c/o Jim Costello, 11 Blackberry Way Penwortham, Preston, Lancashire PR1 9LQ - England Phone: 44 (0)1772 517624, *e-mail:* [info@gorlingroup.co.uk](mailto:info@gorlingroup.co.uk); website: http://www.gorlingroup.co.uk/).

National Basal Cell Nevus Support (NBCCS Support Network) - although no longer a formal organization, Susan Charron will continue to support contacts and informations. (National Basal Cell Nevus Support c/o Susan Charron, 162 Clover Hill Street, Marlboro, MA 01752, (800)-815-4447 or (508)-485-4873, *e-mail*: [Souldansur@AOL.com](mailto:Souldansur@AOL.com)).

BCCNS Patient Advocate – It is a website loaded by Bud Caruso (*e-mail:* [gorlinadvocate@aol.com](mailto:gorlinadvocate@aol.com); Website: http://www.bccns.org/).
